# Assessing attitudes of patient-centred care among students in international chiropractic educational programs: a cross-sectional survey

**DOI:** 10.1186/s12998-019-0263-x

**Published:** 2019-09-12

**Authors:** Karin Hammerich, Kent Stuber, Sheilah Hogg-Johnson, Anser Abbas, Martin Harris, Henrik Hein Lauridsen, Nadège Lemeunier, Michele Maiers, Peter McCarthy, Vanessa Morales, Corrie Myburgh, Vanessa Petrini, Katherine Pohlman, Silvano Mior

**Affiliations:** 1 Canadian Memorial Chiropractic College, 6100 Leslie Street, Toronto, ON M2H 3J1 Canada; 2 Central Queensland University, Rockhampton, Australia; 3 Southern Denmark University, Odense, Denmark; 4 Institut Franco-Européen de Chiropraxie, Toulouse, France; 5 Northwestern Health Sciences University, Bloomington, USA; 6 University of South Wales, Pontypridd, Wales; 7 Parker University, Dallas, USA

**Keywords:** Clinical education, Patient-centred care, Chiropractic students, Surveys and questionnaires

## Abstract

**Background:**

Patient-centred care is internationally recognized as a foundation of quality patient care. Attitudes of students towards patient-centred care have been assessed in various health professions. However, little is known how chiropractic students’ attitudes towards patient-centred care compare to those of other health professions or whether they vary internationally, and between academic programs.

**Objective:**

To assess the association of select variables on student attitude towards patient-centred care among select chiropractic programs worldwide.

**Methods:**

We conducted a cross-sectional study using the Patient-Practitioner Orientation Scale (PPOS) to assess students’ patient-centred attitudes towards the doctor-patient relationship. Eighteen items were scored on a 1 to 6 Likert scale; higher scores indicating more patient-centredness. All students from seven chiropractic educational programs worldwide were invited to complete an online survey. Results were analyzed descriptively and inferentially for overall, sharing and caring subscales. General linear regression models were used to assess the association of various factors with PPOS scores.

**Results:**

There were 1858 respondents (48.9% response rate). Student average age was 24.7 (range = 17–58) years and 56.2% were female. The average overall PPOS score was 4.18 (SD = 0.48) and average sharing and caring subscale scores were 3.89 (SD = 0.64) and 4.48 (SD = 0.52), respectively. There were small but significant differences in all PPOS scores by gender, age, and program. Year/semester of study within a program typically was not associated with scores, neither was history of previous chiropractic care nor having family members who are health professionals.

**Conclusion:**

This is the first international study assessing students’ attitudes of patient-centred care in chiropractic educational programs. We found small but significantly different PPOS scores between chiropractic programs worldwide that did not change across year/semester of study. Scores tended to be lower than those reported among medical students. Observed differences may be related to curricular content, extent of patient exposure and/or regional cultural realities.

## Introduction

Patient-centred care is internationally recognized as a foundation of quality patient care [1]. It is recognized as a key component of the doctor-patient relationship. The World Health Organization [2] defines patient-centred care as an approach to healthcare that is organized around the health needs and expectations of people rather than diseases. A patient-centred approach consciously adopts the perspectives of individuals, families and communities, and sees them as participants as well as beneficiaries of the healthcare system. In such a paradigm, patients have the education and support they need to make decisions and participate in their own care. The World Health Organization’s [2] *“Global Strategy on People-centred Health Services 2016-2026”* outlines a strategy for integrated, people-centred healthcare. Despite its international acceptance, variations in adoption and implementation exist in practice and education [1]. Educating future practitioners is crucial for fostering patient-centred attitudes and behaviours. Student attitudes of patient-centred care have been assessed in various healthcare professions [3–5]. Medicine, nursing and physiotherapy have examined the role of patient-centredness in their respective fields as they relate to chronic illness and musculoskeletal pain in primary care and multi-professional settings [6–10]. To our knowledge, in complementary medicine, the patient care approach has been investigated from the patients’ perspective, but not from the students’.

It is generally accepted measuring all the components of patient-centred care is difficult [11, 12]. However, in evidence-based healthcare, communication, empathy, confidence, knowledge, expertise, professionalism, partnership and health promotion appear to create a positive healthcare experience [8, 9]. In 1996, Krupat et al. [13] developed a measurement tool, the Patient-Practitioner Orientation Scale (PPOS), to assess the attitudes toward doctor-patient relationships as held by doctors and patients. The PPOS examines two dimensions with the sharing component examining attitudes to power, control and sharing of information and the caring component examining attitudes of support and psychosocial aspects. Despite the noted decline in medical students’ attitudes towards patient-centred care as they progress through their program [14], others such as McNair et al. have concluded that “a new generation of doctors with a strong patient-centred focus is emerging” [15]. This focus on patient-centred care is reported [16] in patients seeking care in complementary medicine who experienced greater empathy and empowerment than in conventional medicine.

Studies using the PPOS have investigated a number of factors and their associations to patient-centredness. Cultural effects appear to influence the domains of patient-centredness consistent with the inherent societal norms and preferences of the region [17, 18]. Further, patient-centredness seems to decrease as medical students progress through their training, presumably as they struggle to balance the need to develop self-confidence as doctors while maintaining their motivation to help patients [19, 20]. As Bombeke et al. [21] noted, “despite all educational efforts, the literature shows an ongoing decline in patient-centredness during medical education”.

Other factors reportedly related to patient-centred attitudes of health care students and practitioners are their gender, with females typically scoring higher on average than males [3, 5, 18, 22–24]. Age has also been considered a related variable, with maturity and experiential learning potentially affecting scores [18], although others reported no association [3]. In two other studies of medical students [17, 18], personal experience of continuing care was found to have a positive impact on patient-centred attitudes. On the other hand, the presence of healthcare professionals in the family does not appear to be related to a student’s attitudes toward patient-centred care [3, 17].

The chiropractic literature recognizes the patient-centred paradigm [25–29]. Notwithstanding this recognition in research, healthcare policy, political agendas, and patient satisfaction [12], little is known about how chiropractic educational programs worldwide teach and assess student attitudes of patient-centred care. Such assessment could inform clinical and academic training, as well as implement educational competencies to ensure the provision of quality patient-centred care. Therefore, the purpose of our study was to assess the association of select variables on attitudes of students in international chiropractic educational programs towards patient-centred care.

## Methods

### Design

We conducted a cross-sectional study of chiropractic students during the 2017–2018 academic year using an online survey. The project was approved by the research ethics boards at the Canadian Memorial Chiropractic College (CMCC) (REB# 1404X03) and each participating institution.

### Participants

All students from each participating chiropractic program were eligible to participate. Chiropractic programs were either invited or requested to participate in the study. The programs included the Canadian Memorial Chiropractic College (CMCC) in Canada, Parker University (PU) and Northwestern Health Sciences University (NWHSU) in the United States of America (USA), the University of South Wales (USW) in Wales, the University of Southern Denmark (SDU) in Denmark, L’Institut Franco-Européen de Chiropraxie (IFEC) in France, and Central Queensland University (CQU) in Australia. A total of 3800 students across all institutions were eligible and invited to participate.

### Recruitment and data collection

Participant recruitment followed the Dillman Total Design Survey Method [30]. A 4-week period was allotted for recruitment and data collection. Instructors in mandatory courses were recruited to optimize student participation. Instructors made in-class announcements informing students of the survey at two and one-week intervals prior to initiating the survey. Students were then emailed an invitation with the accompanying link to the survey. A final announcement and invitation were sent to all students 1 week before the survey was closed. Students at PU, CMCC, NWHSU and IFEC were given time to complete the questionnaire during class.

The survey was developed and administered using the online SurveyMonkey platform (SurveyMonkey Inc., San Mateo, California, USA. www.surveymonkey.com). Data extracted from SurveyMonkey were indexed by a study identification number and stored on a secured password protected server located at CMCC. CMCC and IFEC chose to incentivize participants. Those wishing to participate were entered into a random draw for one of four $50.00 gift cards. The email addresses collected for the prize draw were removed before data were extracted for analysis.

## Measures

### Primary outcome

The primary outcome was the Patient-Practitioner Orientation Scale (PPOS) [13]. The PPOS was developed by Krupat et al. [13] to measure respondents’ attitudes toward the doctor-patient relationship along two dimensions, “sharing” and “caring”. The overall average PPOS score range is 1 to 6, where a high overall score indicates that the respondent is patient-centred, while a lower score indicates that the respondent is more doctor-centred. The nine items corresponding to “sharing” measure a respondent’s attitude towards how much power, control, and information should be shared between doctor and patient; the nine corresponding to “caring” measure how much a respondent cares about the warmth and support in a doctor-patient relationship, as well as about a holistic, psychosocial approach to healthcare [13]. Each item is a statement (e.g., “The doctor is the one who should decide what is talked about during a visit”) with response options ranging from “strongly agree” to “strongly disagree”. These options are given numeric values from 1 to 6 with “strongly agree” being assigned 1 for fifteen items and with reversed scoring for the remaining three items. The eighteen items’ values are averaged to get the overall score, and subscale-specific items are averaged to get sharing and caring subscale scores. The PPOS was administered in English for all institutions except IFEC, where a French version [31] was administered. Internal consistency for health care practitioner responders has been shown to be satisfactory for the overall score (Cronbach’s α =0.73 [23]), moderate for the Sharing subscale (Cronbach’s α =0.67 [23] and 0.61 [18]), and moderate for the Caring subscale (Cronbach’s α =0.52 [23] and 0.51 [18]). Validity of the PPOS for health care practitioners is supported by the study by Shaw et al. [32] which showed that practitioners with more patient-centred views had patient encounters with more attention to lifestyle issues, less focus on biomedical matters and more rapport building than practitioners with less patient-centred views.

### Explanatory factors

The primary independent variables of interest were institution and year/trimester of study. Studies suggest that PPOS scores may vary by country of study [3, 18]. We measured PPOS scores among students of programs from different countries. We included the variables, age and sex, which were previously shown to be related to PPOS. Although no relationship was previously seen between medical students’ PPOS scores and whether there were healthcare professionals in the family [3, 17], a relationship was found with personal experience of continuing care [17, 18]. Thus, we wanted to assess if having a family member who is a health care practitioner or experience receiving chiropractic care were related to chiropractic students’ PPOS scores. Some demographic questions were altered to reflect the variations in different institutions, such as year or trimester of study, ethnicity, and previous education level.

### Data analysis

Demographic data were descriptively analyzed, using counts and percentages by category for categorical variables and means and standard deviations for continuous variables. General linear regression models were used to quantify the associations between overall PPOS scores and the independent variables and separately for the Sharing and Caring subscales. The primary independent variables of interest were the institution treated as a fixed effect and year/trimester of study nested within the institution to account for differences in program delivery structure across institutions. We approached modelling in a staged manner. First, the institution and year/trimester of study nested within the institution were included. Then the secondary independent variables age, gender, history of chiropractic care and having family members who are health professionals were added one at a time to examine their significance and whether their inclusion impacted the associations of the primary independent variables. Then a model including all six independent variables was fit. Finally, a model including only those variables with a significant relationship with the outcome was fit.

## Results

Overall, 1961 records were extracted from SurveyMonkey. Of these, we deleted 36 records due to blank or inappropriate responses to demographic questions and 67 records due to missing item responses. Thus, 1858 records across the seven chiropractic programs were used in the analysis (See Table 1).

**Table 1:** Description of numbers (n) of eligible participants, surveys extracted from SurveyMonkey, deleted observations and number of useable surveys

Item	CMCC	PU	NWHSU	SDU	USW	CQU	IFEC	OVERALL
Eligible Students	772	739	601	300	300	242	846	3800
Surveys Extracted	484	368	389	95	84	79	462	1961
Deleted records^a^	7	4	12	2	4	5	2	36
No PPOS Scores^b^	5	15	12	11	3	0	21	67
Useable records	472 (61%)	349 (47%)	365 (61%)	82 (27%)	77 (26%)	74 (21%)	439 (52%)	1858 (49%)

^a^Some records had the same response value for every question in the survey. Some gave answers to demographic questions that were inappropriate

^b^No PPOS scores were calculated due to missing responses to all questions

Response rates varied by program ranging from 25.67 to 61.14%, with an overall response rate of 48.89%. Overall, 57% of respondents were female, 57% were 20 to 24 years of age inclusive, and almost 50% had a family member in healthcare (See Table 2).

**Table 2:** Response rates and demographics of respondents across institutions

Variables	CMCC	PU	NWHSU	SDU	USW	CQU	IFEC	OVERALL
Program length	4 years	10 trimesters	10 trimesters	5 years	4 years	5 years	6 years	
Eligible Students	772	739	601	300	300	242	846	3800
Useable Responses	472(61%)	349 (47%)	365 (61%)	82 (27%)	77 (26%)	74 (21%)	439 (52%)	1858 (49%)
Sex^a^								
Female	280(59%)	154 (44%)	176 (49%)	51 (62%)	43 (56%)	46 (63%)	298 (68%)	1048 (57%)
Male	191(40%)	192 (55%)	186 (51%)	30 (27%)	34 (44%)	27 (27%)	141 (22%)	801 (43%)
Other	1 (0.2%)	2 (1%)	0 (0%)	1 (1%)	0 (0%)	0 (0%)	0 (0%)	4 (0%)
Age^a^								
17–19	0 (0%)	0 (0%)	0 (0%)	0 (0%)	9 (12%)	15 (21%)	60 (4%)	84 (5%)
20–24	291(62%)	155 (45%)	209 (58%)	48 (59%)	44 (57%)	21 (29%)	292 (67%)	1060 (57%)
25–29	161(24%)	121 (25%)	112 (21%)	30 (27%)	11 (4%)	9 (13%)	79 (8%)	523 (28%)
30–34	14 (3%)	34 (10%)	26 (7%)	3 (4%)	7 (9%)	5 (7%)	6 (1%)	95 (5%)
35+	6 (1%)	36 (10%)	16 (4%)	0 (0%)	6 (8%)	22 (21%)	1 (0%)	87 (5%)
Year/Trimester^a^								
1	154(23%)	52 (15%)	127 (25%)	19 (23%)	18 (23%)	24 (22%)	81 (8%)	
2	134(28%)	44 (13%)	0	18 (22%)	13 (17%)	24 (22%)	95 (22%)	
3	86 (8%)	80 (23%)	31 (9%)	17 (21%)	20 (26%)	14 (19%)	58 (13%)	
4	98 (21%)	36 (10%)	75 (21%)	12 (15%)	26 (24%)	9 (12%)	69 (16%)	
5		40 (11%)	0	15 (19%)		3 (4%)	66 (15%)	
6		14 (4%)	29 (8%)				70 (16%)	
7		12 (3%)	51 (4%)					
8		36 (10%)	0					
9		30 (9%)	40 (11%)					
10		4 (1%)	11 (3%)					
Hx Chiropractic Care^a^								
Yes	363(77%)	251 (72%)	321 (88%)	37 (45%)	52 (68%)	68 (93%)	270 (62%)	1362 (74%)
No	108(23%)	96 (28%)	43 (12%)	45 (55%)	25 (22%)	5 (7%)	169 (29%)	491 (27%)
Family Member								
Chiropractor	31 (7%)	28 (8%)	45 (12%)	6 (7%)	9 (12%)	2 (3%)	20 (5%)	141 (8%)
Other HCP	172(26%)	152 (44%)	143 (29%)	38 (46%)	26 (24%)	28 (28%)	178 (41%)	737 (40%)

^a^n (column %)

Internal consistency of the overall PPOS score, Sharing and Caring subscales from these data were Cronbach’s α of 0.67, 0.63, and 0.50 respectively which are similar to values reported by Krupat et al. [23] and Lee et al. [18].

The average overall PPOS score was 4.18 (SD 0.48), with institutional averages ranging from 4.06 to 4.31. The average score for the Sharing component of the PPOS was 3.89 (SD 0.64), and institutional averages ranged from 3.68 to 4.09. The average score for the Caring component was 4.48 (SD 0.52), with institutional averages ranging from 4.25 to 4.75. (See Table 3). Within each institution, there were no significant differences in scores over year or trimester of study except for the overall PPOS score and only for one North American program where the highest means scores were for trimesters 1 and 10 and the lowest mean score for trimester 7 – e.g., the relationship between PPOS score and trimester was U-shaped.

**Table 3:** Mean (SD) PPOS Scores by Institution

	CMCC	PU	NWHSU	SDU	USW	CQU	IFEC
PPOS Score by Year / Trimester	*N* = 472	*N* = 349	*N* = 365^a^	*N* = 82	*N* = 77	*N* = 74	*N* = 439
1	4.21 (0.46)	4.10 (0.49)	4.23 (0.47)	4.00 (0.42)	4.08 (0.42)	4.31 (0.67)	4.14 (0.47)
2	4.33 (0.50)	4.13 (0.63)	–	4.02 (0.34)	4.25 (0.50)	4.41 (0.46)	4.26 (0.40)
3	4.30 (0.46)	4.02 (0.48)	4.21 (0.40)	4.22 (0.41)	4.09 (0.40)	4.30 (0.47)	4.23 (0.42)
4	4.28 (0.41)	4.20 (0.64)	4.06 (0.53)	4.10 (0.50)	4.21 (0.43)	4.00 (0.50)	4.22 (0.41)
5		4.10 (0.47)	–	4.17 (0.57)		4.31 (0.16)	4.17 (0.41)
6		3.85 (0.42)	4.06 (0.35)				4.30 (0.43)
7		4.15 (0.69)	4.01 (0.40)				
8		3.94 (0.49)	–				
9		3.96 (0.55)	4.10 (0.46)				
10		4.18 (0.15)	4.25 (0.28)				
Avg PPOS Score	4.27 (0.46)	4.06 (0.53)	4.13 (0.46)	4.11 (0.45)	4.15 (0.43)	4.31 (0.54)	4.22 (0.43)
Avg Caring	4.50 (0.48)	4.25 (0.55)	4.33 (0.48)	4.55 (0.47)	4.40 (0.49)	4.53 (0.54)	4.75 (0.46)
Avg Sharing	4.05 (0.61)	3.86 (0.68)	3.94 (0.59)	3.68 (0.62)	3.91 (0.63)	4.09 (0.69)	3.70 (0.61)

^a^ Mean Overall PPOS scores for NWHSU are significantly different by Trimester of Study with *p*-value =0.04 from one-way ANOVA. There were no year or trimester differences for any of the other institutions

When institution and year/trimester of study within the institution were included in the models, there was a significant main effect for the institution for all three scores (See Table 4). However, year of study did not significantly contribute to the models for any of three outcomes. When added to the model, age and gender had significant β coefficients for all three outcomes, while history of chiropractic care and having family members who are health professionals consistently did not have significant β coefficients. In addition, their inclusion in the model did not markedly change the β coefficients of institution or year/trimester. Therefore, the final models presented in Table 4, include the independent variables of institution, age and gender.

**Table 4:** Regression models for Overall PPOS score, Caring Subscale, Sharing Subscale

Overall PPOS Score	β	s.e.	95%CI	F/t	df	*p*-value
Intercept	3.87	0.06	(3.75, 4.00)	t = 59.66	1834	<.0001
Institution						
CMCC	ref	–	–	F = 9.69	6, 1834	<.0001
PU	−0.22	0.03	(−0.29, − 0.16)			
NWHSU	−0.14	0.03	(−0.20, − 0.07)			
SDU	−0.18	0.06	(− 0.29, − 0.08)			
USW	−0.11	0.06	(− 0.22, − 0.00)			
CQU	−0.03	0.06	(− 0.15, 0.09)			
IFEC	−0.03	0.03	(−0.09, 0.03)			
Age	0.013	0.002	(0.008,0.018)	F = 29.10	1, 1834	<.0001
Gender						
Female	0.14	0.02	(0.10, 0.18)	F = 41.54	1, 1834	<.0001
Male	reference	–	–	–		
Caring Subscale	β	s.e.	95%CI	F/t	df	*p*-value
Intercept	4.10	0.07	96, 4.23	t = 60.29	1834	<.0001
Institution						
CMCC	ref	–	–	F = 30.12	6, 1834	<.0001
PU	−0.25	0.03	(−0.32, −0.18)			
NWHSU	− 0.16	0.03	(− 0.23, − 0.10)			
SDU	0.04	0.06	(−0.07, 0.15)			
USW	−0.09	0.06	(−0.21, 0.02)			
CQU	−0.02	0.06	(−0.14, 0.10)			
IFEC	0.27	0.03	(0.20, 0.33)			
Age	0.013	0.002	(0.01, 0.02)	F = 26.20	1, 1834	<.0001
Gender						
Female	0.14	0.02	0.10	F = 38.83	1, 1834	<.0001
Male	reference	–	–			
Sharing Subscale	β	s.e.	95%CI	F/t	df	*p*-value
Intercept	3.65	0.09	48, 3.82	t = 41.80	1834	<.0001
Institution						
CMCC	reference	–	–	F = 13.86	6, 1834	<.0001
PU	−0.19	0.04	(−0.28, −0.11)			
NWHSU	−0.11	0.04	(−0.19, −0.02)			
SDU	−0.41	0.07	(−0.55, − 0.26)			
USW	−0.13	0.08	(−0.28, 0.01)			
CQU	−0.04	0.08	(−0.19, 0.12)			
IFEC	−0.33	0.04	(−0.42, − 0.25)			
Age	0.013	0.003	0.007,0.0	F = 16.53	1, 1834	<.0001
Gender						
Female	0.14	0.03	0.08, 0.20	F = 22.43	1, 1834	<.0001
Male	reference	–	–			

In the final model, while institution, age and gender were significant in all three models, the variation in PPOS scores across these variables was small. For instance, spread in mean scores by institution from highest to lowest was 0.22 points (CMCC compared to PU), 0.52 points (IFEC compared to PU) and 0.41 points (SDU compared to CMCC) on a 1 to 6 scale for the Overall, Caring and Sharing scores respectively. Translating these spreads to an effect size metric (dividing by pooled SD of the scores) [32] gave effect sizes of 0.46, 1.0 and 0.64 respectively which can be considered moderate to large as per Cohen’s guidelines (1988). For all three outcomes, the scores were 0.13 points higher on average for each year of increasing age of respondent, and female respondents on average scored 0.14 points higher than male respondents on all three outcomes. For instance, after adjusting for age and institution, the mean overall PPOS score for females was 4.23 (95%CI 4.20–4.27) while for males it was 4.09 (95%CI 4.05–4.13). Diagnostic checks for the three final models (examination of residual plots) suggested model assumptions of normality and common variance were met.

## Discussion

Our findings suggest that chiropractic students’ attitudes tend toward a patient-centred approach to care delivery across international chiropractic programs. However, scores differed significantly between institutions and although these differences were small on the metric of the scales, they translated to having moderate to large effect sizes [33]. In our study, scores increased with increasing student age and were higher on average for females than for males. The overall average PPOS score for chiropractic students in our study was 4.18, with average Sharing and Caring scores of 3.89 and 4.48 respectively. The overall average PPOS score in our study appears to be at the lower end of those reported for medical students, where average scores ranged from 4.1 [18] to 4.66 [34]. Similarly, average Caring subscale scores ranged from 4.4 [18] to 5.20 [34], and average Sharing subscales from 3.8 [18] to 4.10 [34] among medical students. It is of note that the spread of the outcomes measured (as indicated by the standard deviations) appear very similar in these studies, ranging about 0.4 to 0.5. The observed lower score among chiropractic students may be related to curricular content or timing/nature of actual patient exposure in chiropractic programs that primarily occurs in the final term. Such limited exposure may not provide sufficient time for students to develop their self-confidence as doctors nor nurture their desire to help patients, similarly reported among medical students [19, 20].

This is the first study assessing worldwide student attitudes towards patient-centred care in chiropractic education. The Council on Chiropractic Education International aims to harmonize chiropractic education worldwide; however, its advice to national councils are implemented at the discretion of the educational institutions who create curricular content [35]. In our mobile society, common frameworks could ensure consistency across major curricular content worldwide. We observed significant differences across institutions, but it is not clear what might be driving those differences. Woloschuk et al. [14] suggested that examining the hidden curriculum and the null curriculum would identify content that may influence scores of student attitudes. However, our study did not address specific curricula or, arguably more importantly, recruitment policies. As a consequence, it is unknown if and how curricula or recruitment strategy influence student patient-centred attitudes. For example, communication skills are considered a means to seek common ground in the interaction [36], but it is unknown if and how these skills are integrated into the curricula of chiropractic programs, or if/how this capability affects success at the recruitment stage.

Unlike results from other healthcare programs, student PPOS scores in chiropractic programs did not significantly differ by program year or semester. The similar PPOS scores between chiropractic program year or semester may be explained by the curricular content or varying response rates. The varying response rates may also explain the difference between scores reported in medical programs and in our study. Future work could assess how patient centred care is taught across the curriculum and may change over time. In medical school and residency programs, evidence suggests [14, 20, 36] scores degrade over time suggesting a shift towards a more doctor-centred attitude, particularly in Overall and Sharing scores.

The overall tendency for female students to have more patient-centred attitudes is reflective of previous studies [14, 5, 20, 36]. Such higher scores may be due to differences in early socialization, as well as a greater importance placed on empathy specifically for female students and physicians [5]. The tendency for female healthcare students and professionals to communicate in a more patient-centred manner has been reported [14, 5] to affect patient outcomes such as satisfaction. Greater understanding of the development of gender-based differences in patient-centredness may thus have a significant future effect on doctor-patient relations by reducing the gender disparity in patient-centred communication.

Our study identified a positive relationship between student age and PPOS scores, but no relationship between PPOS scores and whether a family member was a healthcare practitioner or not. This is not wholly consistent with previous studies, however, with Haidet et al. [3], reporting similarly with respect to family background, but contrary to us in terms of age and PPOS scores in fourth-year students. Additionally. Lee et al. [18] suggested that a student’s experience of health care systems, either as a patient or having to care for family was associated with higher PPOS scores, but exposure to an acute event was not, attributing the higher scores to maturity gained through life experiences. We, on the other hand, found no relationship between PPOS scores and whether the respondent had received chiropractic care in the past or not.

### Strengths and limitations

A strength of our study was using an instrument which has been used extensively across disciplines and in a context-specific manner that is, testing students’ attitudes. A further strength was that we explored student attitudes across different chiropractic programs worldwide.

Even though the multi-site study was international and used the English version of the PPOS instrument in all schools except one (i.e. IFEC in France), it is unknown if the culture and language of the country affected the understanding and interpretation of the questions. No exploration into cross-cultural adaptation and validation was performed. Additionally, while seven schools participated in this study, although a reasonable guide, the results may not be representative of all chiropractic education worldwide. We did not assess the representativeness of our sample to that of the student body in each program. Thus, the results may not be generalizable to worldwide views of patient-centredness.

Another limitation was our response rate and the risk of non-response bias, which may provide results different than for the entire target student population. Unfortunately, it was not possible to compare responders to non-responders to inform representativeness of the sample. Further, response rates differed across institutions, where three had rates below 33%, which may influence our results; however, institutional response rates did not seem to correlate with PPOS scores. Furthermore, the overall participation rate was recorded as 48.9%, similar to the 43% average online survey response rates reported by Nulty [37].

Finally, despite standardizing our data collection method, there were variations related to administration of the survey, including providing class time and incentives, which may have contributed to differential non-response bias. Indeed, we note that the response rates in the schools providing class time and incentives tended to be higher than those not. To what degree these differences influenced PPOS score is unknown.

The study was conducted across all years and semesters of each chiropractic program. However, no detailed curricular analysis was conducted, hence it is unclear if course content and curricular design/delivery may have impacted the results. Nor did we consider student recruitment policies which may impact results.

## Conclusions

Our study contributes the first data on students’ perceptions of patient-centred care in chiropractic educational programs. We found small but significantly different PPOS scores between chiropractic programs worldwide that did not change across year/semester of study. Future research could explore if such differences are related to program curricula, response bias or if chiropractic student attitudes and their conceptualization of patient-centred care influences patient satisfaction and health care outcomes in the formidable year of clinical training.

## Data Availability

All data generated and analyzed during this study are included in the published article.
